# Social Interaction in Public Spaces and Well-Being among Elderly Women: Towards Age-Friendly Urban Environments

**DOI:** 10.3390/ijerph19020746

**Published:** 2022-01-10

**Authors:** Yingyi Zhang, Ge Chen, Yue He, Xinyue Jiang, Caiying Xue

**Affiliations:** School of Architecture and Urban Planning, Beijing University of Civil Engineering and Architecture, Beijing 100044, China; 201801040220@stu.bucea.edu.cn (G.C.); 201801040221@stu.bucea.edu.cn (Y.H.); 201801040207@stu.bucea.edu.cn (X.J.); 201801040219@stu.bucea.edu.cn (C.X.)

**Keywords:** social interaction, public space, elderly women, urban environment, age-friendly

## Abstract

The world’s population is aging and becoming more urbanized. Public space in urban areas is vital for improving the health of the elderly by stimulating social interaction. Many urban design projects are advertised as age-friendly but ignore the real needs of the elderly, especially elderly women, for social interaction in urban public spaces. Insufficient attention is paid to the physical and psychological characteristics of elderly women when shaping public space. This analysis addresses the question: What are the qualities of urban spaces which facilitate health-improving social interaction for elderly women? Methods include a case study in Beijing, field investigation, mapping, and qualitative and quantitative analysis. The survey was carried out in April 2021, and concerned 240 women aged 55–75 years. Results indicate that the social interactions of older women relate to both their physical and psychological situations. Public spaces can positively impact the psychological well-being and social participation of elderly women. Conclusions include insights regarding the relationship between social interaction and well-being among elderly women, as well as proposing a series of principles for shaping public spaces for an age-friendly urban environment.

## 1. Introduction

Well-being among elderly citizens is a significant contemporary theme. According to the World Health Organization (WHO), population aging and urbanization are two global trends that, together, are major forces shaping the twenty-first century [1]. Urban growth and an aging population form the crux of the current global demographic [2]. Like the broader population, the world’s elderly citizenry continues to become more urbanized [3]. Increasingly, urban research is paying attention to the health of elderly people. Health encompasses more than the absence of disease—mental and social well-being are significant contributing factors. Preserving and developing biological, physiological, and mental functions, as well as social activity, as the population ages is a dynamic process [4]. Health includes being recognized and valued in a social context. Social interaction comprises unacknowledged rituals, tacit understandings, covert symbolic exchanges, impression management techniques, and calculated strategic maneuverings [5]. Face-to-face interaction of even the simplest sort is a socially intricate operation [5]. Public space is an important sphere in which people can maintain a sense of connectivity and social engagement in their later years. It can provide psychological comfort and motivate social behavior. Former research indicates that an age-friendly city offers a supportive environment that enables residents to grow older actively within their families, neighborhoods, and civil society and offers extensive opportunities for their participation in the community [6,7].

Several approaches to defining aging exist in prior research. Chronological age, like sex and race, is a means by which individuals automatically categorize others [8,9]. Cues to age include physical appearance, like hair and facial morphology, as well as verbal and nonverbal communication [10,11,12]. The WHO lends credence to the rising number of residents aged 60 years or over [13]. Neugarten distinguished the “young-old” (i.e., individuals between 55 and 75 years old) and the “old-old” (i.e., individuals 75 years old and older) [14]. He finds stereotypes about the elderly arise from observing the “old-old”, and that these stereotypes are overgeneralized to the “young-old” [14,15]. Numerous studies demonstrate that older adults are not always perceived as a homogeneous group [15,16,17,18,19,20,21]. This paper focuses on the “young-old”, who predominantly make up the active-elderly with relatively good health and who frequently use public space in urban areas.

Urban public space can be an elderly-friendly environment, suitable for addressing and accommodating the challenges facing an aging population. Some projects, however, ignore the real needs of the elderly—notably elderly women. Gender differences show that, beyond the biological differences, it is culture with its social structure including division of gender-related roles, societal functions and social status that has been a more influential factor in determining gender differences in quality of life [22]. 

Elderly women have distinct physical and psychological features. They generally have as little as 37–68% of the muscle strength of males [23]. In later life, men can gradually lose an inch in height; women may lose about two inches [24]. Loss of height results in a lower center of gravity, making it difficult for elderly women to control physical balance. Biological, behavioral, and environmental factors all contribute to the global higher life expectancy of women [25]. Men die younger in all countries [26,27]; elderly women have more problems performing the activities of daily living and have a higher risk of inflammatory-related diseases [28]. In addition, elderly women often suffer more psychological pressure, a sense of inferiority, and alienation than men [29]. According to Wang’s research, the proportion of urban elderly women who feel lonely in social situations is up to 23.09%. Conversely, the same holds true for about 16.86% of urban elderly men [29]. This indicates that elderly women experience more negative social psychology than their male peers. 

Aging is a complex process, encompassing both personal and social factors [30]. Social health includes interpersonal ties and the degree to which individuals are involved in their community [31]. Outside activities are beset with challenges for elderly people, who encounter more hazards than the younger population [32]. On an accessibility front, once spaces are designed to accommodate the elderly, they become more user-friendly for all groups [33]. Conceptualizing public spaces as “places of aging” facilitates a clear encouragement for the elderly to broaden their spheres beyond private spaces. This is a vital opportunity to promote health through the benefits of outdoor activity as part of a daily routine [34]. Qualitative studies have shown that older adults and people with disabilities have higher demands on accessibility (width and surfaces when using walking aids, ramps and railings), orientation, visual relationship, speed (crossings, reaction time) and resting possibilities [35,36]. 

**Figure 1:**
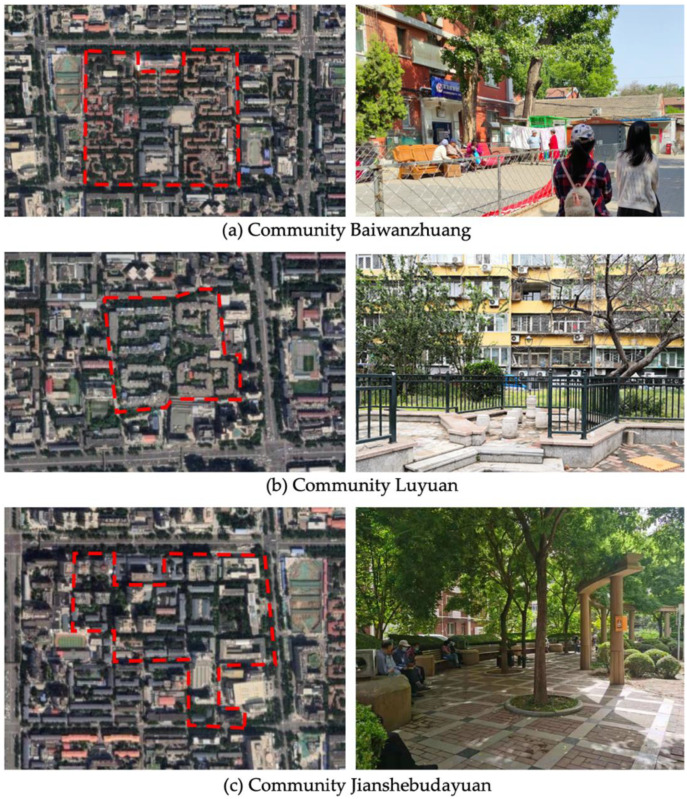
Maps and photos of communities.

Beijing is the site of this case study. The capital of China, the city is home to more than 21 million residents and 16 urban, suburban, and rural districts [37]. As of 2020, the elderly population (above 65) in Beijing has risen to 2.9 million [38]. Around 4.4 million population age between 55 and 75—about 21% of the total population [38]. With such a high-density, elderly population, it is vital to explore how the public space is shaped in the metropolis. Three communities of Beijing have been targeted: Community Baiwanzhuang, Community Luyuan, and Community Jianshebudayuan. Community Baiwanzhuang and Luyuan are both located at the West City District. This district covers the western half of the old city and is home to approximately 1 million inhabitants. High-density communities, historical streets, and modern commercial plazas form the district’s physical environment. Community Jianshebudayuan is situated in the Haidian District. Haidian is the education center of the city, consisting mostly of universities and research institutions. Community Baiwanzhuang was constructed in the 1950s. It is filled with twisting lanes and small-scale neighborhoods. The houses are laid out in strange and complicated patterns, somewhat akin to a labyrinth [39]. Communities Luyuan and Jianshebudayuan were both constructed in the 1990s. They represent relatively modern communities with modern urban design and space shaping modalities. The rationale behind the case study selections includes three aspects. Firstly, the selected communities are located in the densely populated districts of Beijing City. The trend of population aging in the districts is on the rise [38]. The communities can work as typical cases to explore older adults’ well-being. Secondly, according to the latest Detailed Regulatory Plan for the Functional Core Area of the Capital (Block Level) (2018–2035), the selected communities are included in the priority renewal areas [40]. Thirdly, public spaces of the selected communities are relatively old and staleness. There is an urgent need to improve quality to shape an age-friendly urban environment. Figure 1 presents the photos of Community Baiwanzhuang, Community Luyuan and Community Jianshebudayuan.

This paper focuses on social interaction in public spaces and the associated well-being of elderly women. It uses a Beijing case study to address the requirements of, and challenges to, social interaction for elderly women in public space. The research has three major goals: (i) an analysis of the types and characteristics of the social interactions of elderly women; (ii) the relationship between requirements for effecting social interaction for elderly women and effective public space shaping; (iii) outlining principles for guiding public space shaping towards an age-friendly urban environment. The research question asks: What are the qualities of urban spaces which facilitate health-improving social interaction for elderly women? Accordingly, the potentiality of public space as contributing to the age-friendly urban environment is analyzed, assessing the benefits of social interaction for the well-being of elderly women.

## 2. Materials and Methods

Around 240 elderly women aging between 55 and 75 from three communities in Beijing participated in the field survey. There are 5812 households in the communities in total, including 2089 households in Community Baiwanzhuang, 2127 households in Community Luyuan, and 1596 households in Community Jianshebudayuan [41]. In Community Baiwanzhuang, elderly population (above 55 years old) is around 1692 [37], and more than 100 older adults are above 90 years old [42]. In community Luyuan, elderly population (above 55 years old) is around 1722 [37]. In Community Jianshebudayuan, the elderly population (above 55 years old) is around 1292 [37]. The survey took place over 2 weeks between 1 and 15 April 2021. 

**Figure 2:**
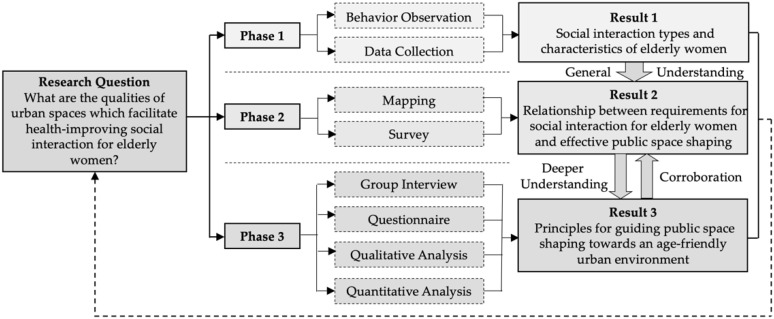
Methodological framework.

The methodological framework is comprised of three phases (Figure 2). Phase One consisted of behavior observation and data collection from the three Beijing communities targeted for observation. Observing behavior enabled a general understanding of social interaction behaviors of elderly women in the three communities. The elderly women’s ages were asked to identify observation targets aging between 55 and 75. Data collection from observation during Phase One recorded the time and interactions happening in public spaces. The results were indicative of the types and characteristics of social interaction among elderly women. According to the general understanding of elderly women’s social interaction behaviors, major public spaces were chosen to be investigated in Phase Two. The second phase covered mapping for the survey. Selected public spaces, such as multi-scale squares and green gardens, were mapped and in order to analyze the social interaction routines of elderly women. The result reflected the relationship between social interaction requirements of elderly women and public space shaping. In order to corroborate observations and mapping of the first and second phase, the survey between local elderly women was launched in Phase Three. Phase Three consisted of group interviews, questionnaires, and qualitative and quantitative analysis. Elderly women’s willingness and preferences for public space shaping were asked and analyzed. The result was three principles that can effectively guide public space shaping towards an age-friendly urban environment. The authors collected data in each phase. 

### 2.1. Observation and Data Collection

Observation and data collection served to gain an understanding of the social interactions of elderly women within the nominated communities. Observation is the process of scrutinizing and describing the social interaction behavior of elderly women. It was a method of collecting information of watching elderly women’s activities and recording it for analysis in a later phase. During field observation in the nominated communities, social interaction type, place, period, and a detailed description of each community were noted in recording sheets. Data collection was another significant step in this phase. Data summarizing the number of elderly women who enter a public space for social interaction, frequency of social interaction, and distances between home and public space, were collected in order to analyze the characteristics of social interaction behaviors. 

### 2.2. Mapping

The purpose of the second phase was to reveal the relationship between requirements for social interaction of elderly women and public space shaping. Many types of public space may provide places for social interaction in the nominated communities. Mapping facilitates an understanding of the public space’s site selections, design, and shaping effects. Mapping tools utilized included scale plate, digital level, and a drawing implement. Once the public space measurement data were collated, a series of computer-aided plans could be generated with digital software. Developed and marketed by Autodesk company [43], AutoCAD is a computer-aided design and drafting software running on microcomputers with internal graphics controllers [44]. AutoCAD was used to generate digital plans with internal graphics controllers. 

Survey methods were used to study a randomly selected sample of older women in the nominated communities by talking with them and recording their activities. Through posters, flyers and inquiries, participants learned about the purpose and content of the survey. They could take part in the survey by filling out questionnaires or verbal communication. The surveys were semi-structured, enabling a mix of qualitative and quantitative data to be collected. Topics included whether participants made full use of the public space, their most and least favorite elements of the public space, and their willingness to spend time with friends there. The survey results identify ways in which the design of public space affects elder women’s enthusiasm for social interaction. 

### 2.3. Survey and Analysis

Interviews and questionnaires were the survey techniques used to explore the principles that could guide public space shaping towards an age-friendly environment. Interviewing is a qualitative research technique that involved asking open-ended questions to facilitate conversation with the elderly women of the sample communities. Interviews were unstructured, with a thematic approach rather than a rigid question structure. 

Questionnaires were used to understand the satisfaction of elderly women with current public spaces and comprehend their conception of a public space ideal for social interaction. Valid returned questionnaires consisted of 96 from Community Baiwanzhuang, 89 from Community Luyuan, and 55 from Community Jianshebudayuan. Valid returned questionnaires of Community Baiwanzhuang were 40% from various communities, Community Luyuan produced 37% and Community Jianshebudayuan produced 23%. Invalid returned questionnaires consisted of 4 from Community Baiwanzhuang, 11 from Community Luyuan, and 5 from Community Jianshebudayuan. According to Beijing Municipal Bureau of Statistics, elderly women populations number about 846 in Community Baiwanzhuang, 861 in Community Luyuan, and 646 in Community Jianshebudayuan [37]. It means around 12% of the elderly women in Community Baiwanzhuang, 12% in Community Luyuan, and 9% in Community Jianshebudayuan answered the survey.

The questionnaires addressed a range of themes and included closed- and open-ended survey questions. The content consisted of three sections. 

First was basic information, including age, health status and family composition. The purpose of collecting basic information was to understand situations of questionnaire participants. 

Second was social interaction activities in participants’ leisure times of day, including when leisure times were, how long they stayed in public space, what activities they usually did, what kind of facilities in public space they preferred, and which public space they used. The purpose was to understand elderly women’s specific social interaction preference.

Third were requirements of and satisfaction with public space, including Likert Scale questions related to atmosphere satisfaction of different public spaces, enthusiasm, engagement, and social participation motivation. The purpose was to understand what kinds of public space can be friendly and accessible to elderly women. 

Respondents answered the questionnaire anonymously. Data security and privacy were protected and used exclusively for the purpose of this academic research. Ethical approval was given officially. 

Data collected through interview and questionnaires were analyzed in this phase. Quantized data, such as time, number of elderly women using public space, number of elderly women who chose different social interaction activities, were statistically summed up and expressed as bar graphs, pie graphs, and line graphs. Descriptive data, such as elderly women’s feedbacks about enthusiasm and motivation, were summarized as guiding principles for public space design. 

## 3. Results and Discussion

### 3.1. Social Interaction of Elderly Women

**Figure 3:**
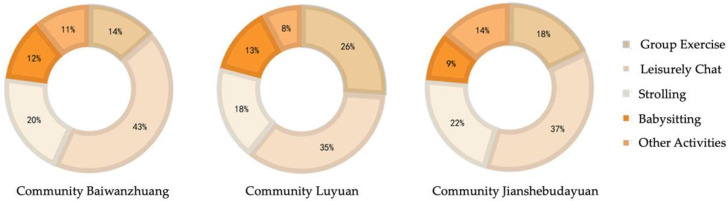
Types and proportions of social interaction of elderly women.

Results from observations (types of interaction) and questionnaires (period/times, public places) indicate that most of the social activities of elderly women are family-centered. Their interactions took place largely in the small-scale squares and green places of their neighborhoods, which form the primary public space for elderly women’s communication. Social interaction types included group exercise (such as square dancing), leisurely chatting, strolling, babysitting, and other activities (such as religious gatherings). Data from the questionnaires and interviews (Figure 3) illustrate the percentage rates of social interaction types within the three communities. In Community Baiwanzhuang, 43% of survey participants preferred to chat with friends and neighbors in public spaces. Strolling (20%) and group exercise (14%) were also popular. The elderly women of Community Luyuan spent more time chatting for leisure (35%). Group exercise took second place (26%) followed by strolling (18%). In Community Jianshebudayuan, elderly women preferred chatting (37%) and strolling (22%). Group exercise also featured (18%), as did other activities (14%), and babysitting (9%). In-keeping with Wang’s statement (2016), the investigate data indicated a preference for gentle activities, such as chatting or strolling, over more tiring ones. Wang also found that urban elderly women, some 88.08% of the questionnaire participants, had an interest in communicating with others [29]. The same was true for 80.15% of rural elderly women.

**Figure 4:**
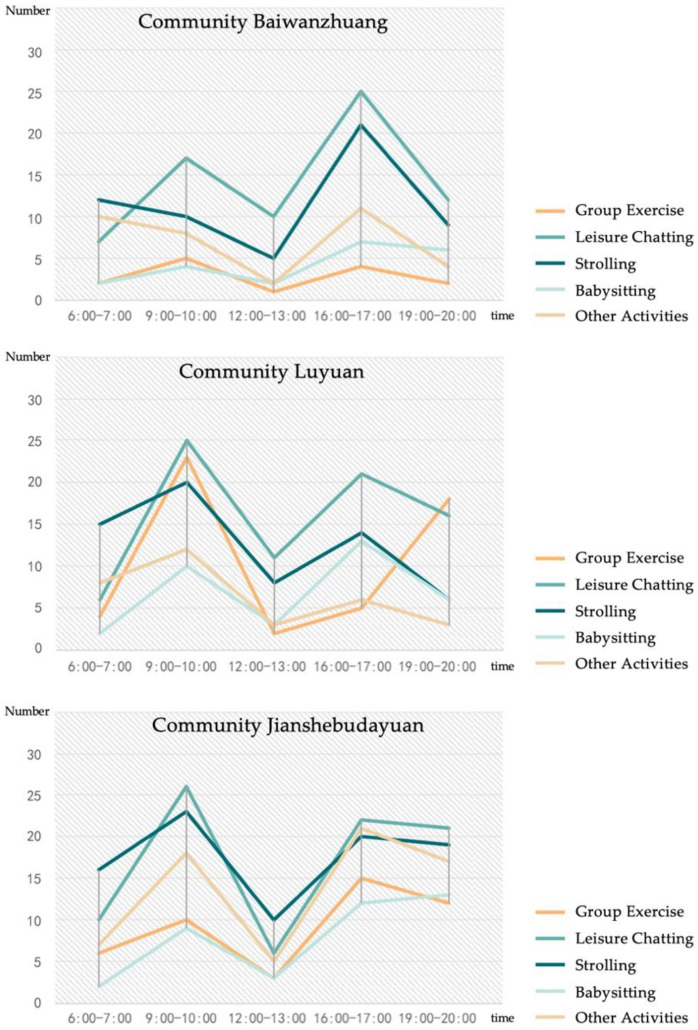
Time and types of social interaction among elderly women.

Data from observation and questionnaires show the preferred time of social interaction. Elderly women in the sample communities predominantly interacted socially during mornings and afternoons. As Figure 4 indicates, 09:00 to 10:00 and 16:00 to 17:00 were peak periods of social interaction. In Community Baiwanzhuang, about 17 elderly women chatted in a public space between 09:00 and 10:00, and 25 from 16:00 to 17:00. In the early morning, most elderly women liked to stroll with friends. Few chose to interact socially in public spaces at noon. From late afternoon until evening, elderly women engaged in chatting, strolling, and babysitting. In Community Luyuan, elderly women preferred to chat, stroll, and do group exercises from 09:00 to 10:00. In the evening, they tended to do group exercises, such as square dancing and martial arts, as well as engage in leisurely talk. Babysitting occupied the smallest proportion of a day. Chatting and strolling in the morning were also popular in Community Jianshebudayuan. Twenty-six elderly women chatted in a public space from 09:00 to 10:00 and 22 between 16:00 and 17:00. Twenty-three elderly women strolled with friends or neighbors from 09:00 to 10:00 and 20 at 16:00 until 17:00. Group exercise, babysitting and other social activities frequently happened from 16:00 to 20:00. These data indicate that elderly women tend to stay indoors during the mid-day period. 

**Figure 5:**
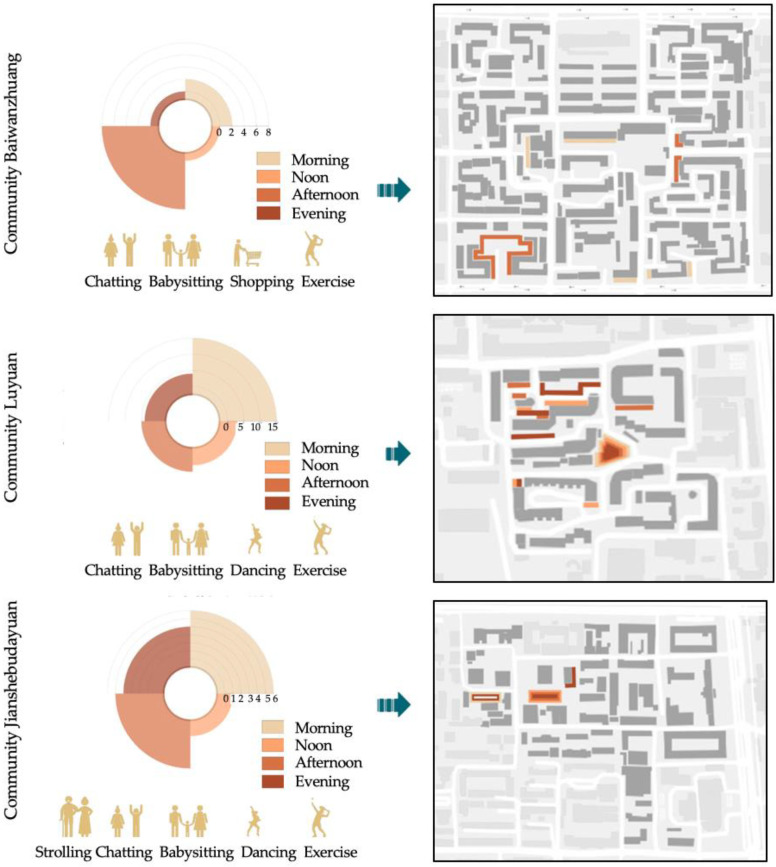
Social interaction among elderly women and public space sites.

Survey results also reveal that elderly women preferred familiar places for social interaction. Public spaces are expected to be safe environments which meet the physical and psychological security needs of elderly women. Many people chose to age in a place where they used to live, preferring social interactions in a familiar, secure environment. The US Center for Disease Control and Prevention defines aging in place as the ability to live in one’s own home and community safely, independently, and comfortably, regardless of age, income, or ability level [45]. As Wiles et al. indicate, “aging in place” was seen as an advantage in terms of a sense of attachment or connection and feelings of security and familiarity in relation to both homes and communities [46]. The data from observations (Figure 5) depict the primary locations where the elderly women of the sample communities interacted socially. Clear areas and small-scale squares around residential buildings were the most common choice of elderly women. In Community Baiwanzhuang, elderly women liked to chat or babysit close to their own houses. In addition, the square in the south-west corner of the community functions as the public center of the community. On average, eight elderly women interacted socially in this space in the afternoons. Community Luyuan features a central square in the middle of the community. Social interactions happened in this square from morning to night. Areas in the northern area of the community also provide public space for social interaction, especially from afternoon to evening. In Community Jianshebudayuan, elderly women liked to socially interact in the small square in the west area of the community from the afternoon until evening. The small square is a stimulus for social activities.

Observations in the communities reflect that social interactions within the sample communities can be grouped into three distinct categories. The first of these is basic social interaction, including, for example, leisurely chatting and babysitting. These interactions happen largely around houses and closed yards, in green places between residences, and in the entrances of houses and alleys. Elderly women might walk about 10 min to arrive at the target public space. Communication is predominantly between families, relatives, and close friends. The second category is neighborhood social interaction. Interactions in this category include strolling and gentle group exercises. They occur in outdoor squares, green areas, or public centers. Elderly women might walk about 20 min to arrive at their destination. These social interactions occur most often in the afternoon and evening, and communication is commonly between neighbors. The third is an extension of neighborhood social interactions. Representative interaction types include group exercises such as square dancing, martial arts, and ball games. Elderly women may need to walk or take public transportation to arrive at the public space in about 30 min. Social interaction in this category may be between strangers. Elderly women may experience anxiety about leaving home and negotiating access to extend the scope of their neighborhood social interaction.

### 3.2. Public Space for Elderly Women’s Social Interaction

**Figure 6:**
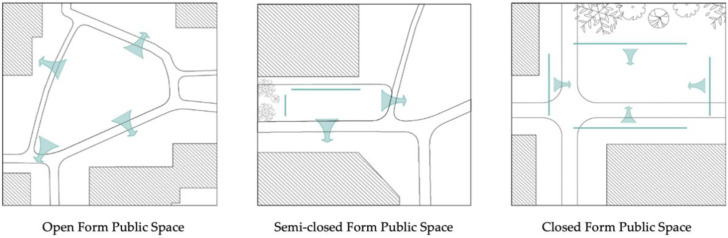
Social interaction among elderly women and public space sites.

Three forms of public space provide places for elderly women’s social interaction. These are: open form, semi-closed form, and closed form (Figure 6). Open form public spaces, like that in the mid-area of Community Luyuan, can be surrounded by roads. They preserve scenic views of the adjacent landscape. Elderly women tend to access this form of public space for group exercises or chatting. Flexible usage and the large population capacity of this type encourages social activities. Semi-closed form public spaces exist in all sample communities. They may be located between two buildings and an open place. The surfaces of adjacent buildings partially conceal the public space, enabling a measure of privacy. As a result, intimate conversations between close friends may happen in this form of public space. Closed form public spaces are usually small-scale and surrounded by buildings or greenery. They provide a sheltered space in which elderly women can sit for a while or communicate with family, friends, or neighbors. Although typically close to houses and easily accessible, these forms have less of a direct line of sight so may present a security risk to older women. 

A selection of public spaces within the sample communities was analyzed. The central square of Neighborhood Mou, and the open areas of Neighborhood Chen and Neighborhood Zhongli in Community Baiwanzhuang, were evaluated for public space shaping and social interaction behaviors. Table 1 illustrates that both formal public spaces, such as the central square, and informal public spaces, like open areas within neighborhoods, can be sites of social interaction. Elderly women use different spaces in different ways. Group exercises with others take place most often in formal spaces, while casual chatting and gentle exercise with friends is more frequently seen in informal spaces. Existing fitness equipment in these spaces is perceived as dangerous by some elderly women, who consider it appropriate mainly for young adults. 

In Community Luyuan, the central square, and the clear and corner areas of Neighborhood Nanluyuan have been selected for analysis. Observations and mapping data are summarized in Table 2. Elderly women favor the community central square for group exercise. Square dance is a favorite of elderly women, and the central square is usually a large, paved open area. Smaller scale open spaces such as Neighborhood Nanluyuan’s clear area are more suited to babysitting and chatting. Elderly women prefer briefer communication in informal public spaces adjacent to residential buildings (for example, the corner area of Neighborhood Nanluyuan). 

Community Jianshebudayuan features public space shaping which incorporates botany (Table 3). Most public space here is surrounded by greenery. Elderly women consider this community to have quality public space for social interaction. They prefer to chat, stroll with friends, or do group exercises in the green space. There is a strong requirement for community life among the elderly women, and connections between them and the wider society are diverse. They might be smiling or saying hello in the small square, or chatting about shared interests. Public space with greenery and infrastructure is beneficial for communication and facilitates a sense of community and citizenship among elderly women. 

### 3.3. Principles of Age-Friendly Urban Space Shaping

As a result of this research into the communities of Beijing, including the interviews with elderly women, we propose three principles of age-friendly urban space shaping. These principles apply specifically to “young-old” elderly women who are aged between 55 and 75. This demographic generally enjoys better health than “old-old” elderlies and are thus more eager to engage with their communities. 

The first principle is one of security. It is essential ensure safe public spaces exist for outdoor social interactions. Older people, whether they are retired or working from home, spend a much larger proportion of their time within the residential environment than do younger people [47]. Accordingly, public spaces in residential environment are the main social interaction places for the elderlies. Encompassing more than age-friendly infrastructures, the security principle addresses psychological security for elderly women. For example, public space shaping can avoid creating remote corners. Green belts should not be so long or high that they obscure sight. Allowing elderly women to observe their surroundings without obstacles helps them decide if a space is safe. The research clearly demonstrates that elderly women prefer to attend square dances in the evening. As a result, evening lighting is necessary around public squares. 

Convenience is the second principle. Some elderly women reflect that social interaction in the community is a source of worry. Noisy environments, motorways, and a lack of pedestrian crossings prevent older women from venturing outside. Brach and VanSwearingen indicates, in older adults, walking is slow, less stable, inefficient, and the timing and coordination of stepping with postures and phases of gait is poor [48]. This research is accordance the statement by observing elderly women’s daily social interactions. Improved walking systems for the elderly are thus important in the process of public space shaping. Squares or green spaces are best if they provide barrier-free access for elderly women. Seats are also required as the elderly may need a rest anytime, anywhere.

The third principal is comfort. Small-scale, landscaped spaces are appreciated by elderly women. They like chatting with friends and neighbors and enjoying sunlight in small-scale spaces. A comfortable atmosphere facilitates a relaxed social psychology, encouraging social interaction. Greening should avoid allergen or flocculent plants.

Social interaction gives elderly women a sense of citizenship. In the urban design field, the voices of older people are often treated as secondary and are rarely integrated into plans for urban regeneration and physical transformation [49]. Public space in urban areas can be places of comfort for elderly women as they continue to age in the community. 

## 4. Conclusions

Quality public spaces in urban areas are a significant opportunity to encourage the elderly to interact socially. This research highlights that public space should address elderly women’s special social interaction behaviors, needs and experiments. Through analyzing how elderly women interact and behave in the public spaces of the communities in Beijing, it was found that social interaction, such as talking, strolling with friends, and group exercises, is an essential part of the lives of elderly women. One of elderly women’s needs is maintaining communication with others to avoid loneliness. This is in accordance with the findings of Wang that more elderly women feel lonely in later life than elderly men [29]. Elderly people differ from the younger generation who tend to be more confident and independent in a social occasion. Temelova and Slezakova (2014) also indicated how public spaces should be used is different among different generations [50,51].

This research argues that general public space shaping guidelines and standards cannot fully incorporate the social interaction requirements of elderly women. Just offering a separate blank open place in current urban design projects may fail to meet the broad spectrum of needs of different genders and ages. Public spaces suitable for elderly women are diverse. Social interaction may take place in central squares, in clear areas between residences, and at corner side entrances. Formal and informal public space can provide a variety of atmospheres to motivate a range of social interactions. Public spaces with greenery, pavements, seats, and fitness equipment attract elderly women, who come to meet with friends, relatives, and neighbors. 

It is a challenge for urban planners, designers and architects to establish comfortable environments that are inclusive and caring to its residents, especially to the elderly people [51]. By field investigating in communities, it is found comfortable environments make elderly women confident to access facilities. A strong sense of community is generated, and community life becomes active if the public space caters to the needs of all age and gender groups. Public space that is safe, convenient, and comfortable can facilitate social interaction for elderly women and thus improve their health. Principles of security, convenience, and comfort generate elderly women-friendly public space. They support the social network enhancement and well-being of elderly women and stimulate participation and interaction in elderly women’s later lives.

## Figures and Tables

**Table 1:** Public space observation and mapping of Community Baiwanzhuang.

Public Space	Plan	Characteristics	Infrastructure	Photos
Central square of Neighborhood Mou	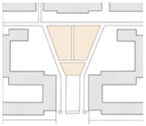	Concentrated open space Direct connections to surroundings both visual and physical Vibrant activities	Ping pong tables Benches Fitness equipment	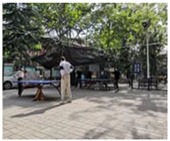
Clear area of Neighborhood Chen	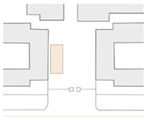	In front of residential buildings Close to the community entrance Convenient access Dull space shaping	Benches	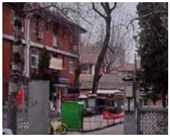
Clear area of Neighborhood Zhongli	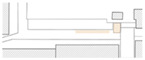	Informal public space Convenient access Used by close neighborhoods	Benches	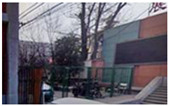

**Table 2:** Public space observation and mapping of Community Luyuan.

Public Space	Plan	Characteristics	Infrastructure	Photos
Central square of Community Luyuan	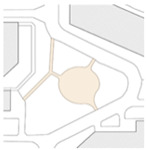	Concentrated open space Direct connections to surroundings both visual and physical Vibrant activities	Wood gallery Benches	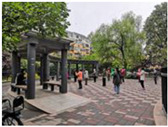
Clear area of Neighborhood Nanluyuan	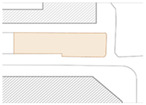	In front of residence buildings Conveniently accessed Low outdoor thermal comfort during daytime	Benches Fitness equipment	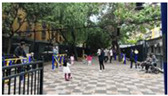
Corner area of Neighborhood Nanluyuan	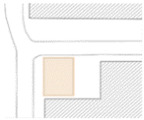	Informal public space Conveniently accessed Used by close neighborhoods	Benches	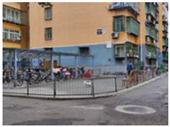

**Table 3:** Public space observation and mapping of Community Jianshebudayuan.

Public Space	Plan	Characteristics	Infrastructure	Photos
Green space of No.3 Residence Building	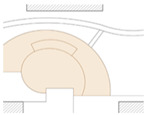	Formal open space with large scale Direct connections to surroundings both visual and physical Vibrant activities	Wood gallery Benches	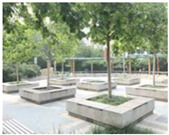
Green space of No.7 Residence Building	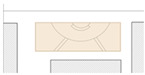	In front of residence buildings Conveniently accessed Close to secondary road	Wood gallery Benches Chinese chess table	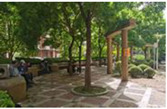
Small square aside Property Management Office	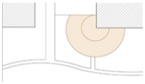	Conveniently accessed Close to secondary road	Fitness equipment Sandpit	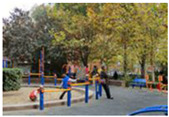

## Data Availability

Data sharing is not applicable to this article.

## References

[1] World Health Organization (WHO) (2007). Global Age-Friendly Cities: A Guide.

[2] Phillips D., Siu O., Yeh A., Cheng K., Andrews G.J., Phillips D.R. (2005). Ageing and the Urban Environment. Ageing and Place: Perspectives, Policy, Practice.

[3] Alidoust S., Holden G., Bosman C. (2014). Urban Environment and Social Health of the Elderly: A Critical Discussion on Physical, Social and Policy Environments. Athens J. Health.

[4] Koipysheva E.A., Lebedinsky V.Y., Koipysheva M.A. (2018). Physical Health (Definition, Semantic Content, Study Prospects). Proceedings of the International Conference on Research Paradigms Transformation in Social Science.

[5] Little W. (2015). Introduction to Sociology.

[6] Caro F.G., Fitzgerald K.G. (2016). International Perspectives on Age-Friendly Cities.

[7] Hoof J.V., Kazak J.K., Perek-Bialas J.M., Peek S.T.M. (2018). The Challenges of Urban Ageing: Making Cities Age-Friendly in Europe. Int. J. Environ. Res. Public Health.

[8] Brewer M.B., Srull T.K., Wyer R.S. (1988). A Dual-process Model of Impression Formation. Advances in Social Cognition.

[9] Fiske S.T., Gilbert D.T., Fiske S.T., Lindzey G. (1998). Stereotyping, Prejudice, and Discrimination. The Handbook of Social Psychology.

[10] Bieman-Copland S., Ryan E.B. (2001). Social Perceptions of Failures in Memory Monitoring. Psychol. Aging.

[11] Hummert M.L., Garstka T.A., Shaner J.L. (1997). Stereotyping of Older Adults: The Role of Target Facial Cues and Perceiver Characteristics. Psychol. Aging.

[12] Montepare J.M., Zebrowitz-McArthur L. (1988). Impressions of People Created by Age-related Qualities of their Gaits. J. Personal. Soc. Psychol..

[13] Alves F.B., Mendes L.T. (2012). Urban Design and Ageing-public Space for Elderly People in Residential Areas, Proceedings of Planning and Aging—Think, Act and Share Age-Friendly Cities, Porto, Spain, 31 January 2012.

[14] Neugarten B. (1974). Age Groups in American Society and the Rise of the Young-old. Ann. Am. Acad. Political Soc. Sci..

[15] Richeson J.A., Shelton J.N., Carstensen L.L., Hartel C.R. (2006). A Social Psychological Perspective on the Stigmatization of Older Adults. When I’m 64.

[16] Braithwaite V., Gibson D., Holman J. (1986). Age Stereotyping: Are We Oversimplifying the Phenomenon?. Int. J. Aging Hum. Dev..

[17] Brewer M.B., Dull V., Lui L.L. (1981). Perceptions of the Elderly: Stereotypes as Prototypes. J. Personal. Soc. Psychol..

[18] Brewer M.B., Lui L.N. (1984). Categorization of the Elderly by Elderly: Effects of Perceiver’s Category Membership. Personal. Soc. Psychol. Bull..

[19] Hummert M.L. (1990). Multiple Stereotypes of Elderly and Young Adults: A Comparison of Structure and Evaluations. Psychol. Aging.

[20] Hummert M.L. (1994). Physiognomic Cues and the Activation of Stereotypes of the elderly in Interaction. Int. J. Aging Hum. Dev..

[21] Schmidt D.F., Boland S.M. (1986). Structure of Perceptions of Older Adults: Evidence for Multiple Stereotypes. Psychol. Aging.

[22] Carmel S. (2019). Health and Well-Being in Late Life: Gender Differences Worldwide. Front. Med..

[23] Chen G., Liu L., Yu J. (2012). A Comparative Study on Strength between American College Male and Female Students in Caucasian and Asian Populations. Sport Sci. Rev..

[24] Ventura Orthopedics. https://venturaortho.com/do-people-really-shrink-with-age/.

[25] Ortiz-Ospina E., Beltekian D. Why Do Women Live Longer Than Men?. https://ourworldindata.org/why-do-women-live-longer-than-men.

[26] Barford A., Dorling D., Smith G.D., Shaw M. (2006). Life Expectancy: Women Now on Top Everywhere: During 2006, Even in the Poorest Countries, Women Can Expect to Outlive men. BMJ.

[27] Leon D.A. (2011). Trends in European Life Expectancy: A Salutary View. Int. J. Epidemiol..

[28] Crimmins E.M., Shim H., Zhang Y.S., Kim J.K. (2018). Differences between Men and Women in Mortality and the Health Dimensions of the Morbidity Process. Clin. Chem..

[29] Wang L. (2016). An Analysis on Elderly Women and Social Service Demands. J. China Women’s Univ..

[30] Clarke P., Nieuwenhuijsen E.R. (2009). Environments for healthy ageing: A critical review. Maturitas.

[31] Donald C.A., Ware Jr J.E., Brook R.H., Davies-Avery A. (1978). Conceptualization and Measurement of Health for Adults in the Health Insurance Study: Vol.V, General Health Perceptions.

[32] Fietkau J., Stojko L. (2020). A System Design to Support Outside Activities of Older Adults Using Smart Urban Objects, Proceedings of 18th European Conference on Computer-Supported Cooperative Work, Siegen: Germany, 14–17 June 2020.

[33] Patil D.R., Raj M.P. (2021). Designing Urban Public Spaces for Walkable Mobility of Elderly Residents: A Model of Assessment & Strategic proposals-Case of Bangalore City, India. Proceedings of the 8th Zero Energy Mass Custom Home International Conference.

[34] Peace S., Kellaher L., Holland C. (2005). Making Space for Identity Critical Approaches to Ageing and Later Life.

[35] Moran M., Cauwenberg J.V., Hercky-Linnewiel R., Cerin E., Deforche B., Plaut P. (2014). Understanding the Relationships Between the Physical Environment and Physical Activity in Older Adults: A Systematic Review of Qualitative Studies. Int. J. Behav. Nutr. Phys. Act..

[36] Bruchert T., Baumgart S., Bolte G. (2021). Social Determinants of Older Adults’ Urban Design Preference: A Cross-sectional Study. Cities Health.

[37] Beijing Municipal Bureau of Statistics Beijing Municipal Bureau of Statistics and NBS Survey Office in Beijing. http://nj.tjj.beijing.gov.cn/nj/main/2019-tjnj/zk/indexeh.htm.

[38] National Bureau of Statistics 7th National Census. http://www.stats.gov.cn/ztjc/zdtjgz/zgrkpc/dqcrkpc/.

[39] Keats Baiwanzhuang. https://keatschinese.com/china-culture-resources/baiwanzhuang/.

[40] Beijing Municipal Commission of Planning and Natural Resources Detailed Regulatory Plan for the Functional Core Area of the Capital. http://ghzrzyw.beijing.gov.cn/zhengwuxinxi/ghcg/xxgh/sj/202008/t20200829_1993379.html.

[41] Anjuke Beijing Households in Communities of Beijing. https://beijing.anjuke.com.

[42] Beijing News Three Generations of Aunts in Xicheng District. https://baijiahao.baidu.com/s?id=1646074738226574989&wfr=spider&for=pc.

[43] Autodesk, Inc. History. http://www.fundinguniverse.com/company-histories/autodesk-inc-history/.

[44] Autodesk, Inc. Chapter 8: Autodesk and AutoCAD. http://cadhistory.net/08%20Autodesk%20and%20AutoCAD.pdf.

[45] Centers for Disease Control and Prevention Healthy Places Terminology-Aging in Place. https://www.cdc.gov/healthyplaces/terminology.htm.

[46] Wiles J.L., Leibing A., Guberman N., Reeve J., Allen R.E.S. (2012). The Meaning of “Aging in Place” to Older People. Gerontologist.

[47] Sarkissian W., Stenberg B. Guidelines for Planning for Older People in Public Open Space. https://sarkissian.com.au/wp-content/uploads/sites/13/2013/09/Older-people-in-residential-public-open-space.pdf.

[48] Brach J.S., VanSwearingen J.M. (2014). Interventions to Improve Walking in Older Adults. Curr. Transl. Geriatr. Exp. Gerontol. Rep..

[49] Center for Aging Better Putting Older People at the Heart of Urban Transformation. https://ageing-better.org.uk/blogs/putting-older-people-heart-urban-transformation.

[50] Temelova J., Slezakova A. (2014). The Changing Environment and Neighborhood Satisfaction in Socialist High-Rise Panel Housing Estates: The Time-Comparative Perceptions of Elderly Residents in Prague. Cities.

[51] Yung E.H.K., Conejos S., Chan E.H.W. (2016). Social Needs of the Elderly and Active Aging in Public Open Spaces in Urban Renewal. Cities.

